# Severe Acute Bronchial Asthma with Sepsis: Determining the Status of Biomarkers in the Diagnosis of the Disease

**DOI:** 10.3390/diagnostics13162691

**Published:** 2023-08-16

**Authors:** Susmita Sinha, Santosh Kumar, Mahendra Narwaria, Arya Singh, Mainul Haque

**Affiliations:** 1 Department of Physiology, Khulna City Medical College and Hospital, 33 KDA Avenue, Hotel Royal Crossing, Khulna Sadar, Khulna 9100, Bangladesh; 2 Department of Periodontology, Karnavati School of Dentistry, Karnavati University, Gandhinagar 382422, Gujarat, India; 3 Asian Bariatrics Plus Hospital, V Wing-Mondeal Business Park, SG Highways, Ahmedabad 380054, Gujarat, India; 4 The Unit of Pharmacology, Faculty of Medicine and Defence Health, Universiti Pertahanan Nasional Malaysia, Kuala Lumpur 57000, Malaysia; 5 Department of Scientific Research Center (KSRC), Karnavati School of Dentistry, Karnavati University, Gandhinagar 382422, Gujarat, India

**Keywords:** asthma exacerbation, septicemia, biomarkers, disease prognosis, procalcitonin, C-reactive protein, FeNO, blood eosinophil

## Abstract

Bronchial asthma is a widely prevalent illness that substantially impacts an individual’s health standard worldwide and has a significant financial impact on society. Global guidelines for managing asthma do not recommend the routine use of antimicrobial agents because most episodes of the condition are linked to viral respiratory tract infections (RTI), and bacterial infection appears to have an insignificant impact. However, antibiotics are recommended when there is a high-grade fever, a consolidation on the chest radiograph, and purulent sputum that contains polymorphs rather than eosinophils. Managing acute bronchial asthma with sepsis, specifically the choice of whether or not to initiate antimicrobial treatment, remains difficult since there are currently no practical clinical or radiological markers that allow for a simple distinction between viral and bacterial infections. Researchers found that serum procalcitonin (PCT) values can efficiently and safely minimize antibiotic usage in individuals with severe acute asthma. Again, the clinical manifestations of acute asthma and bacterial RTI are similar, as are frequently used test values, like C-reactive protein (CRP) and white blood cell (WBC) count, making it harder for doctors to differentiate between viral and bacterial infections in asthma patients. The role and scope of each biomarker have not been precisely defined yet, although they have all been established to aid healthcare professionals in their diagnostics and treatment strategies.

## 1. Introduction

More than 300 million people worldwide have asthma, which poses an alarming danger to public health. Bronchial asthma is a non-communicable disease that affects a person’s standard of living and psychological and physical wellness. The consequences of this illness can be severe and continue throughout the patient’s life, influencing caregivers, family members, societies, and the healthcare sector. A precise diagnosis of this condition is essential to track the health of those with severe asthma. The recently developed clinical biomarkers have been determined to be a practical tool for disease diagnosis in the clinical management of critical illness. Even though all biomarkers have been developed to assist healthcare providers in their diagnostic and therapeutic approaches, their specific roles and scope are still unclear. This review aimed to provide insight into the various biomarkers of severe acute asthma with sepsis and determine their prognostic implications.

The progression of an asthmatic episode in severe acute asthma typically occurs over days or weeks, though in a few individuals, it may happen over hours or even minutes. This condition is potentially fatal and is one of the “gateways” of access to asthmatic deaths [1]. The diagnosis of severe acute asthma must be made at the emergency room since it might rarely appear as an entirely novel problem in a patient unaware of asthma [2]. Therefore, morbidity and mortality are primarily caused by underestimating the severity of the exacerbation, delaying hospital referrals, and/or providing insufficient emergency care. Acute bronchial asthma is a paramount global healthcare concern regarding complications, death [3,4], and economic impact. Episodes of coughing (especially at night or in the earlier mornings), dyspnea, wheezing, or tightness in the chest that are linked to an extensive but varying airflow restriction inside the lung are referred to as severe episodes of asthma [5]. This condition is frequently recoverable either spontaneously or with medical treatment. In general care and emergencies, it is the source of a significant percentage of antibiotic prescriptions [6]. Until now, antibiotic treatment has not been advised for viral and bronchial infections. Antibiotics are recommended when there is a high-grade fever, a consolidation on the chest radiograph, and purulent sputum that contains polymorphs rather than eosinophils [7]. Bronchial asthma is caused by lower respiratory tract inflammation and bronchial smooth muscle spasms that are usually mediated by IgE.

Since there are currently no feasible clinical or radiological signs that can easily distinguish between viral and bacterial infections, managing acute bronchial asthma with sepsis is difficult. Again, the specific decision about whether or not to commence antimicrobial treatment remains challenging [8]. The role and scope of each biomarker have not been precisely defined yet, even though they have all been established to aid healthcare professionals in their diagnostics and treatment strategies [9,10,11]. Since viral infections are typically involved in asthma attacks brought on by a medical condition, antibiotics are not recommended as a standard treatment [12,13].

Various inflammatory reactions with clinical suspicion or confirmation of a microbial etiology are called sepsis [14,15]. Again, sepsis continues to be a significant cause of morbidity and death and a global concern in various therapeutic contexts, despite rising acknowledgment of its significance [16,17]. A simple bedside or fast laboratory evaluation with highly accurate traits that could distinguish a bacterial cause that requires antimicrobial medication from a nonbacterial cause would be crucial for advising treatment involving the start and stoppage of antimicrobial agents [18,19].

Cellular and organ functioning has been evaluated using biological markers, often known as “biomarkers,” along with the spectrum of wellness and disease [20]. The goal of biomarkers is to guide medical professionals in identifying the bacterial or viral cause of acute respiratory infection to minimize or at least reduce the requirement for antimicrobial medication [21].

As markers of airway or systemic inflammation, fractional exhaled nitric oxide (FeNO), blood eosinophil counts (EOS), and neutrophil-to-lymphocyte ratio (NLR) have been used to increase the precision of asthma diagnosis, direct asthma interventions, track the effectiveness of inhaled corticosteroids (ICS) therapy, evaluate eosinophilic airway inflammation, and determine the likelihood of acute exacerbation. FeNO [22], EOS [23], and NLR [24] are efficient, feasible, consistent, and non-invasive inflammatory biomarkers. Even so, elements like smoking and prescribed medicines impact all of them [25,26,27,28].

## 2. Objective of the Study

Various difficulties can be classified as impediments that prevent the accurate diagnosis of sepsis, including patients, medical personnel, and others. Additionally, sepsis is a primary cause of fatalities in intensive care units (ICUs), and it can be hard to foresee the progression of the disease in patients. In the clinical management of critical illness, the recently designed clinical biomarkers have been acknowledged as a feasible tool for diagnosing diseases. The goal of this review was to illuminate the distinct biomarkers of severe acute asthma with sepsis and figure out their prognostic relevance.

## 3. Materials and Methods

**Figure 1:**
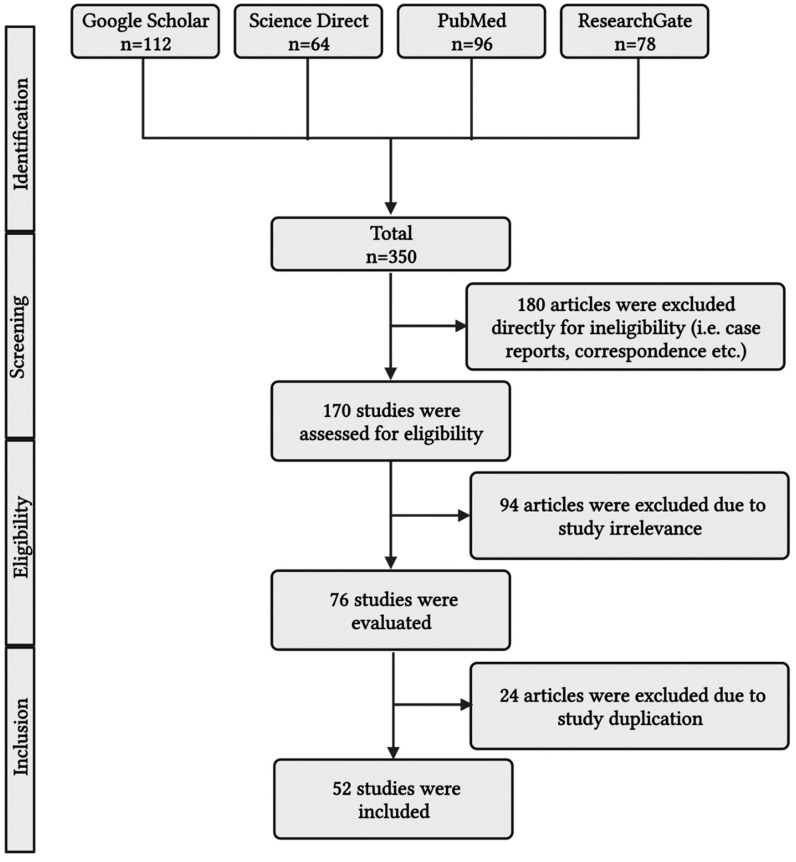
A simplified PRISMA diagram showing the methodology. This figure has been drawn using the premium version of BioRender with the license number LK25NTV1VO. Image Credit: Susmita Sinha.

This article explains common biomarkers for evaluating and managing severe acute bronchial asthma with septicemia. Google Scholar, Science Direct, PubMed, and ResearchGate were among the online archives reviewed for the scientific literature search (Figure 1). The reference list of relevant works was reviewed to retrieve additional materials. Keywords included acute severe bronchial asthma, sepsis, septicemia, biomarkers, bronchial asthma prognosis, procalcitonin, C reactive protein, blood eosinophil in bronchial asthma, neutrophil to lymphocyte ratio, and hydrogen sulfide. Additionally, predominant keywords included: severe bronchial asthma and sepsis, described in Table 1, as PICO format. Keywords also include insulin resistance, polyunsaturated fatty acids (PUFA), omega-fatty acids, and omega-3 fatty acids. Papers written in languages other than English and released prior to 2000 were not included. The papers’ eligibility was thoroughly considered before they were included in the study. Duplicate publications were removed. After the recommended works of literature were independently evaluated and included, an additional discussion was organized to discuss any doubts, issues, inaccuracies, or prejudices relevant to the individual article.

## 4. Diagnosis of Asthma

Incorrect diagnosis could lead to adverse effects from asthma treatments. Therefore, it is crucial to perform a thorough examination to determine whether the patient has severe asthma. The initial step in the diagnostic process is comprehensive history-taking and careful general physical examination [45]. Symptoms, how frequently they occur, and their seriousness must be pointed out in the patient’s medical record. It is also essential to assess the degree of exacerbations and related comorbidities and clarify the onset of symptoms. Diagnosis of severe asthma exacerbations is of the utmost importance since they are associated with adverse effects and involve regular surveillance and intensive management. The history of medications could point to insufficient care or poor adherence to a recommended course of therapy. Socioeconomic factors and the absence of a written asthma action plan are also linked to an increased risk for severe exacerbation [46,47].

## 5. Pathophysiology of Bronchial Asthma

Severe acute asthma requires the most excellent attention, monitoring, and management skill to keep a patient’s asthma from becoming uncontrolled with fatal clinical outcomes. Patients with harsh asthma experience notable troubles with everyday life, such as decreased activity levels, less efficiency at work, and isolation from society. In addition, patients with severe asthma must cope with a higher incidence of complications [48]. Increased resistance to airflow, decreased expiratory flow, the accumulation of air with each breath, and lung hyperinflation are all effects of small airway obstructions within the lungs and make the expiration process an active process [49,50]. Additional mechanical difficulties result from a flat diaphragm resulting from hyperinflation [51]. Forced vital capacity and expiratory volume are reduced while total lung volumes are raised. Total lung capacity (TLC) continues to rise in acute severe asthma exacerbations, aiding in keeping narrowed airways open. At the same time, in physiological conditions, the quiet expiration process occurs passively through the elastic recoil tendency of the lungs. The time required for the inspired tidal volume to expire fully will increase with declined elastic forces, and inspiration starts at a volume when the respiratory system displays a positive recoil pressure due to incomplete exhalation of the given tidal volume. Moreover, positive alveolar pressure at the end of expiration causes the flow, known as dynamic hyperinflation of the lungs [52,53]. 

**Figure 2:**
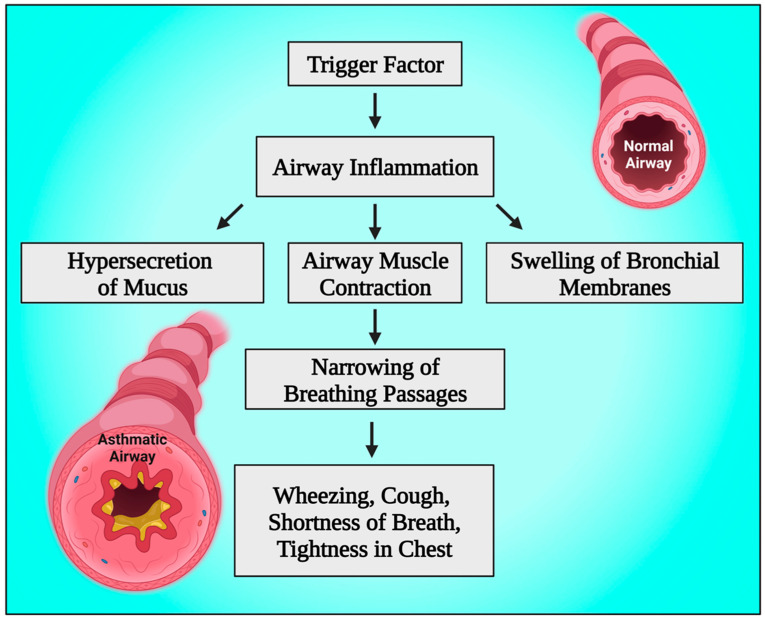
Schematic diagram showing the pathophysiology of bronchial asthma. This figure has been drawn using the premium version of BioRender with the license number GD25JNBBY1. Image Credit: Susmita Sinha.

In addition, increased intrapulmonary shunt, increased dead space, and mismatched ventilation-perfusion ratio (V/Q) are the causes of abnormal gas exchange [54,55]. Airway inflammation and smooth muscle constriction, which may sometimes be severe enough to cause a potentially fatal airway obstruction even without mucus plugging, are the primary contributors to decreased airflow [56,57]. Asthma-related inflammation includes airway edema, eosinophilic cellular infiltration, activated CD4+ T lymphocytes and mast cells, and intraluminal mucous plugs made of plasma proteins, mucin glycoproteins, epithelial and inflammatory cells, as well as cellular debris [58,59]. A severe asthma attack that results in considerable dynamic hyperinflation also has another crucial component called hemodynamic impairment. In people with severe asthma, dynamic hyperinflation can stretch the pulmonary vasculature, raising right ventricular afterload and vascular resistance [60,61]. By reducing the proper heart preload and increasing the right heart afterload, the development of positive intrathoracic pressures causes a decrease in the right heart outputs. A significant reduction in systolic arterial pressure during inspiration and the presence of the pulses paradoxes is acknowledged by the decreased right heart output in conjunction with the left heart’s diastolic dysfunction and its incomplete filling [62,63] (Figure 2).

## 6. The Emerging Role of Biomarkers

Biomarkers are observable traits that can be quantitatively assessed to determine if a biological process is typical or pathological. There are four primary functions that biomarkers play in therapeutic settings: (1) diagnostic; (2) disease grading; (3) continual monitoring of the disease’s advancement; and (4) evaluation of the efficacy of treatment. In addition to serving as a clinical reference, the technique of biomarker analysis enables the identification of potential opportunities for innovative therapeutics and an in-depth knowledge of the primary molecular pathways that contribute to disease progression [64].

**Figure 3:**
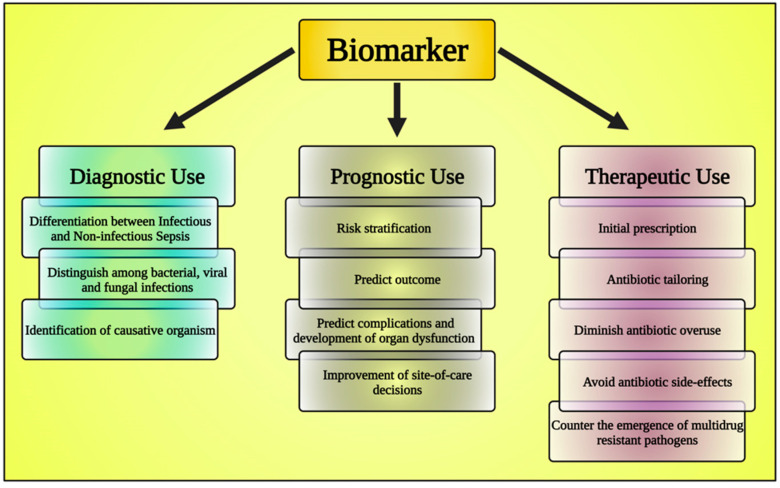
The chart shows the classification of biomarkers. This figure has been drawn utilizing the premium version of BioRender with the license number DA25JLV4IV. Image Credit: Susmita Sinha.

In every aspect of medical management, the value of biomarkers is increasing. Whether used to anticipate, identify, or track health conditions, biomarkers are helpful at every stage of the course of treatment [65] (Figure 3). Biomarkers can be assessed by examining blood, sputum, and urine samples [66,67]. All medical practitioners should understand biomarkers, their uses, and their potential consequences on patient outcomes when biomarker research is implemented into clinical practice [68]. The employment of biomarkers has evolved into an integral component of the standard of care for several illnesses because of considerable research and clinical evidence, which has increased the significance of biomarkers in detecting and managing many diseases [69]. Advancement in the implementation of biomarkers for disease classification, monitoring, and assessment should lead to more effective disease management and enhanced tailoring of therapy [70,71]. 

### 6.1. Asthma Biomarkers

Asthma is a persistent breathing disorder caused by respiratory tract inflammation [72]. Regarding asthma, endotyping and phenotyping are intimately related to biomarkers. The purpose is to anticipate a response to a specific treatment using a variety of signals, either systemic, local, or clinical [73,74]. 

#### 6.1.1. Fractional Exhaled Nitric Oxide (FeNO)

FeNO is the most frequently examined potential biomarker for asthma [75,76]. Nitric Oxide (NO) is produced in the lungs in response to inflammation by the enzyme Nitric Oxide synthase from the amino acid L-arginine [77,78]. Assessing FeNO is simple, rapid, and noninvasive. FeNO values are influenced by height, age, weight, race, gender, and exhaling flow rates [79,80].

#### 6.1.2. Sputum Inflammatory Cell Analysis

The most reliable, precise, and non-invasive tool for determining airway inflammation is sputum inflammatory cell examination, which identifies the many inflammatory phenotypes of asthma [81,82]. Again, its dependability, sensitivity, and authenticity are established, and processing and evaluation are controlled [83]. 

Asthma characteristics have been reported to be related to higher sputum neutrophil levels. In individuals with chronic asthma, prolonged narrowing of the airways and a gradual decline in lung function have been linked to airway neutrophilia. It has also been related to increased bronchial responsiveness unrelated to elevated eosinophil count [84].

Patients with asthma had considerably greater sputum periostin levels than non-asthmatics. Periostin is a potential biomarker capable of identifying intensity and outcome and serving as a possible treatment focus [85]. Sputum periostin has an inverse relationship with forced expiratory volume in the first second (FEV1) and is highly associated with sputum TLC and age. It is linked to both the sputum eosinophil percentage and neutrophil count [86]. Again, periostin is a matricellular protein expressed by lung fibroblast and respiratory epithelium. In addition to this, periostin is a secondary product of type-2 immunological reactions’ distinguishing cytokines, interleukin-13 and interleukin-4 [87].

Th-1 and Th-17 actions are shifted due to neutrophil infiltration and engagement into the respiratory passages, initiating toll-like receptor (TLR) activity and triggering innate immunity. Again, this process produces elevated levels of neutrophil elastase, interleukin-8, matrix metalloproteinase 9, and interleukin-17. IL-8 causes neutrophils to release enzymes and other impacts on immunological monitoring and bacterial death. Neutrophil subgroups may exert diverse effects on immunological monitoring and bacterial death. In addition, bacteria, ozone, and viruses cause the release of cytokines and chemokines that ultimately encourage neutrophil migration [88,89].

#### 6.1.3. Blood Eosinophil (B-EOS)

Eosinophils have been playing a more important part as a biomarker in determining the response to treatment in clinical practice for several years [90]. High B-EOS findings were observed to be a significant indicator of treatment response. Contradictory findings have been found regarding the proper B-EOS cut-off for estimating airway eosinophilia in severe asthma [91,92].

#### 6.1.4. Total Serum IgE Level

In severe asthma caused by allergies, this biomarker guides anti-IgE antibody treatments. Blood eosinophil count (more than 260 per liter) and fractional exhaled nitric oxide (FeNO) values above 19.5 parts per billion strongly indicate whether individuals with severe allergic asthma are responsive to anti-IgE antibodies that will lower exacerbation incidence in asthma patients [22,93,94].

#### 6.1.5. Soluble Form of the Triggering Receptor Expressed on Myeloid Cells-1 (sTREM-1)

The immunoglobulin superfamily member sTREM-1 is represented on the outer membranes of neutrophils, monocytes, and macrophages [95]. Its production is boosted by extracellular bacteria, which also causes the release of cytokines that promote inflammation. Thus, it amplifies the inflammatory response in contact with bacteria [95,96].

#### 6.1.6. Neutrophil to Lymphocyte Ratio (NLR)

NLR could be utilized as a biomarker to differentiate and diagnose various forms of obstructive illnesses since neutrophils and NLR, as markers for circulatory immune complexes, can be significantly more prevalent among individuals with respiratory insufficiency than in the healthy population [97,98].

Healthcare research on early diagnostic markers in asthma patients is an area of interest. The study of readily available biomarkers for the diagnosis of asthma has drawn more attention in the past few years. As an indicator of persistent infection, the blood neutrophil-to-lymphocyte ratio (NLR) is a simple, easily accessible, and reasonably priced index obtained from complete blood counts. The NLR has been considered to be a potential indicator of episodes of inflammation in persistent illnesses and several other conditions in a number of recent studies. In addition, a rise in neutrophils is a result of cytokines in the pathophysiology of asthma. Additionally, patients with asthma exacerbations had greater blood NLR values than those with stable asthma [24,99].

### 6.2. Sepsis Biomarkers

The prognosis of sepsis remains detrimental regardless of the increased use of modern technologies for its management. Again, sepsis is one of the most common causes of death across the world, and its fatality rates are particularly significant because there is no accurate approach for predicting the course of the condition. Since sepsis has a mortality rate between 10% and 50%, managing this medical condition remains complicated. One of the main reasons for fatalities in the ICU is sepsis; hence, sepsis biomarker development and research are of utmost importance [100,101]. Since sepsis shares many clinical signs with other disorders that develop in ICU patients, recognizing the condition can be challenging for medical professionals [102,103]. The two most often utilized indicators for sepsis and other bacterial illnesses are C-reactive protein (CRP) and procalcitonin (PCT) [104,105].

#### 6.2.1. Procalcitonin

Procalcitonin (PCT) is a precursor of calcitonin, consisting of 116 amino acid peptides synthesized in healthy people’s thyroid and adipose tissue [106]. To maintain calcium homeostasis, it is cleaved to produce calcitonin, which is then stored and produced in a controlled manner [107,108]. PCT has a serum value of 0.1 ng/mL in a healthy individual [109]. In addition to this, it was found to be more prevalent in patients with systemic illnesses [110]. Other illnesses, like surgery and trauma, as well as systemic viral infections to a much lesser amount, have also been linked to increased focus. The highest levels of serum PCT are observed in multiorgan dysfunction brought on by trauma and bacterial infection [111,112].

During trauma and surgery, PCT synthesis is induced throughout all parenchymal tissues by the systemic inflammatory response, especially by inflammatory mediators such as tumor necrosis factor-alpha (TNF alpha) [113]. Moreover, in reaction to an illness or damage, procalcitonin rises within 4 h, peaks at 6 h with an 8–24 h plateau, and then falls back to normal within 2–3 days. In contrast, CRP has an onset of 12–24 h, a plateau of 20–72 h, and a return to baseline of 3–7 days or more [114,115].

#### 6.2.2. Prognostic Role of Procalcitonin

Even though PCT is more expensive, researchers have discovered that it helps separate bacterial from noninfectious inflammation causes [116]. Randomized controlled research revealed that serum procalcitonin (PCT) values could efficiently and safely minimize antibiotic usage in individuals with severe acute asthma [117]. Patients with systemic bacterial infections have elevated amounts of PCT in their blood. In contrast, patients with viral infections or inflammatory disorders still have relatively low levels of PCT in their blood. PCT levels may help clinical decision-making regarding the start and end of antibiotic therapy [118,119].

#### 6.2.3. C Reactive Protein

CRP was regarded as a generalized but sensitive indicator of the beginning of inflammation [120]. The liver primarily synthesizes CRP in response to the cytokine interleukin-6, which is secreted during infections and several inflammatory conditions [121]. By attaching to the polysaccharides on pathogens, it begins a complement activation. Although sepsis is under control, its extended half-life indicates that it stays positive for a long time [122].

It is difficult for clinicians in feasible healthcare environments to determine which asthma patients with bacterial respiratory tract infection (RTI) will be effectively treated with antimicrobial therapy [123,124]. Despite current clinical guideline warnings against empiric antibiotic administration in severe asthma exacerbations, patients frequently receive antibiotics [125]. The clinical manifestations of acute asthma and bacterial RTI are similar, as are commonly used test values, like C-reactive protein (CRP) and white blood cell (WBC) count, making it harder for doctors to differentiate between viral and bacterial infections in asthma patients [126]. Due to the increased morbidity and mortality associated with severe exacerbations, patients are more typically managed with antibiotics.

Standard tests performed in laboratories, such as CRP level and WBC count, are frequently used; however, this application appears to be influenced more by traditional practices than by the diagnostic efficacy of these assays [127]. Furthermore, delayed peak values and poor specificity of CRP level and/or WBC count, particularly in individuals with systemic inflammation, limit their usefulness for directing antibiotic treatment [128].

#### 6.2.4. Type 2 Helper T-Cell (Th 2)

Th-2 immune responses, an essential causative process, primarily cause asthma. Increased circulating Th-2 concentrations have been reported in patients who survived than in those who had passed away from Staphylococcus aureus infection [129]. In addition to this Th-2 path, research on nonTh-2 pathways further points towards possible positive effects of asthma for predicting the outcome of infection. Again, toll-like receptors (TLR) are crucial in the allergic response of the respiratory passageways because they are the primary detectors of intruding microorganisms. Furthermore, the pathogenesis of asthma involves the stimulation of interleukin 17 (IL-17), which is possibly significant in causing the attraction of neutrophils to the infection site and, thus, minimizing disease progression [130].

#### 6.2.5. Omentin-1

A newly discovered adipokine with anti-inflammatory characteristics linked to sepsis and inflammatory disorders is omentin 1, also known as intelectin 1. Omentin-1 primarily appears in fat tissues of viscera, although it can be detected in the ovaries, endothelium, the bloodstream, mesothelial cells, and respiratory goblet cells. Again, the concentration of serum omentin-1 rises in sepsis, and the severity and 28-day mortality of sepsis correlate with more significant levels and slower kinetics during the first week of the condition [131].

#### 6.2.6. H_2_S

It has long been established that hydrogen sulfide (H_2_S), a toxic gas with a strong, putrid egg odor that is linked to industrial and water pollution, counts as a harmful gas [132]. In addition to this, the respiratory and central nervous systems are significantly affected by H_2_S. Recent research, however, implies that H_2_S may belong to a unique class of endogenous gaseous transmitters and, along with carbon monoxide and nitric oxide, may constitute a third endogenous signaling transmitter that functions both as a vasodilator and a neurotransmitter [133,134]. Furthermore, it has been found that increased H_2_S production in endotoxemia contributes to the pathogenesis of organ damage [135]. Acting at the junction of leucocytes and endothelium, endogenous H_2_S is a significant facilitator of acute inflammatory processes [136]. In the etiology of sepsis, shock, cardiovascular injury, and pancreatitis, endogenous H_2_S may have anti-inflammatory or pro-inflammatory impacts, indicating that H_2_S may be linked to the development of systemic inflammation [137]. However, the diagnostic usefulness of serum H_2_S in bacterial infection in patients who are not critically ill has not been examined.

## 7. Takeaway Message

Acute severe asthma with sepsis can be treated more rapidly, with better results, and with less needless antibiotic therapy with an early diagnosis. Diagnostic biomolecular markers have the potential to considerably optimize, speed up, and accurately represent the entire recovery process, from diagnosis and management to confirmation and prompt therapeutic adjustment. Procalcitonin (PCT) has variable cut-off limits in various clinical circumstances, although it is still effective for the identification of sepsis in medical settings. Moreover, procalcitonin is an intriguing biomarker for the diagnosis of bacteria-related sepsis since it is capable of distinguishing culture-positive and culture-negative sepsis from non-infectious illnesses [138].

Blood neutrophils may be helpful as sepsis biomarkers. The blood neutrophil-to-lymphocyte ratio (NLR), which is derived from total blood counts, is a simple, easily available, and relatively affordable index used as a marker of chronic infection. In a number of recent studies, the NLR has been proposed as a potential marker of bouts of inflammation in chronic diseases and various other illnesses. Furthermore, patients with asthma exacerbations had greater blood NLR values than those with stable asthma [24,99]. 

**Figure 4:**
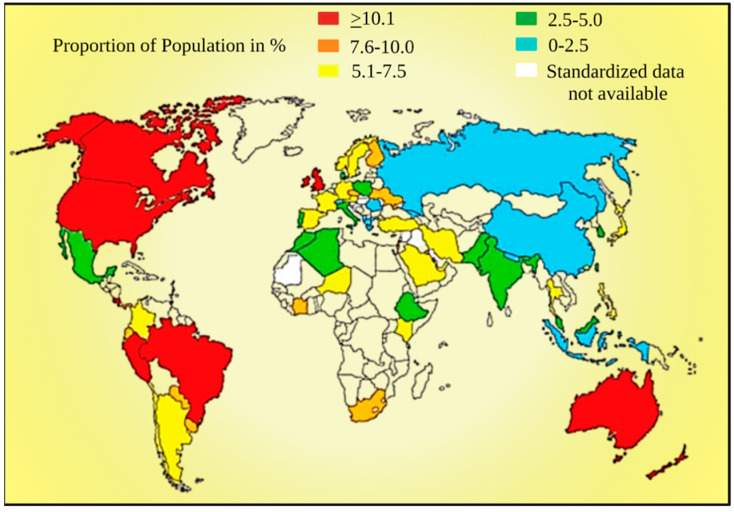
Diagram showing the prevalence of asthma worldwide [150]. This figure has been drawn using the premium version of BioRender with the license number AN25JJ2Y2S. Image Credit: Susmita Sinha.

Early sepsis causes a significant increase in the concentration of CRP, and because of this, it has been employed to diagnose sepsis and its prognosis. Furthermore, the importance of circulating HS-CRP (high sensitivity C-reactive protein) in the diagnosis of asthma is increased when paired with fractional exhaled nitric oxide (FeNO). In contrast, procalcitonin (PCT) is beneficial in the diagnosis of bacterial infections in patients and has an impact on decisions about antibiotic therapy. Most importantly, PCT is capable of distinguishing severe acute asthma from sepsis. Additionally, Pearson correlation analysis revealed that NLR, CRP, and PCT levels were conclusively correlated with graveness of septicemic patients, especially those with bloodstream infections (*r*-values were 0.468, 0.456, and 0.670, respectively; all *p* < 0.001) [139]. Furthermore, multiple studies reported that NLR has been considered the top biomarker to diagnose sepsis [140,141,142,143]. It has been reported that NLR levels are raised in any chronic systemic inflammatory state, including severe acute asthma, cancer, atherosclerosis, and endocrine stress [26,144,145,146]. Multiple studies reported that the integrated result of CRP level and NLR are considered propitious biomarkers for recognizing bronchial asthma [147,148,149]. The global prevalence of bronchial asthma is depicted in Figure 4. 

## 8. Conclusions

It can be challenging for medical professionals to determine the cause of sepsis because it exhibits many clinical symptoms that are similar to other illnesses which might arise in intensive care unit (ICU) patients. The introduction of antimicrobial therapy during lower respiratory tract infections can be guided by the implementation of biomarkers (PCT or CRP), and the excessive use of prescribing antibiotics in health care can be decreased. PCT is a more applicable and accurate biomarker for identifying the bacterial (high PCT) as opposed to viral (low PCT) origin of lower respiratory tract infections than CRP. The advancement of PCT throughout time seems to be associated with patient mortality and prognosis. The primary biomarkers regularly employed in everyday clinical practice for diagnosing, phenotyping, and managing asthma are FeNO, blood eosinophils, and total IgE. The demand for the generation of biomarkers to aid doctors in managing asthma is growing due to the need to more accurately and rapidly phenotype asthma, foresee complications, and determine whether or not interventions are responding.

## Figures and Tables

**Table 1:** Depicted the Principal Keywords Severe Bronchial Asthma and Sepsis in the PICO format [29,30,31,32,33,34,35,36,37,38,39,40,41,42,43,44].

Patient/Problem	Intervention	Comparison	Outcome
Severe Bronchial Asthma	Biomarkers determine the severity of disease and either include or exclude sepsis [29,30,31,32,33].	Clinical identification, evaluation, and therapeutic intervention of severe acute asthma [34,35,36].	Unnecessary use of antibiotics is avoided [37,38,39].
Sepsis	The mortality rate of sepsis decreases when the commencement of focused therapy and therapeutic interventions is delayed. Biomarker assessment may improve discrimination of inflammation from sepsis [40,41,42].	The early goal-directed therapy aims to give early antibiotics to those with infection due to bacteria. Compared biomarker sensitivity to routine care [43]	Rapid detection and implementation of relevant measures may be achieved with biomarkers. In the patient group with bronchial asthma and sepsis, biomarkers improve identification of sepsis [44].

## Data Availability

Information is taken from freely available sources for this review paper.
